# The Work of the Holy Ghost

**Published:** 1887-06-11

**Authors:** 


### Words of Consolation.

[Communications for this column should be addressed, "Chaplain," care of the Editor of THE HOSPITAL, who will gratefully welcome any suggestions or inquiries from matrons, nurses, and the staff generally.]

### XXXVI.—The Work of the Holy Ghost.

We proceed to the consideration of another branch of the Holy Spirit's work. "He will guide you into all truth" (John xvi, 13). The idea of a guide is that of a person who is appointed or invited to show us some way which we have already made up our minds to travel along. If we have our minds set towards heaven; if we are true followers of Jesus Christ along the narrow way, then the Holy Ghost becomes our guide. But He cannot strictly be called our guide until we have deliberately chosen the path of life. We must make up our minds to a journey before we ask for a guide. We must have decided upon a voyage before we call for a pilot. We are therefore probably correct in asserting that the Holy Ghost is only the guide of persons who have already allowed Him to convince them of sin, of righteousness, and of judgment. He may remain for a long time with the sinner pricking him to the heart, stirring the conscience, striving by every means to alter him, but this Divine guidance, strictly speaking, is only for those who have quite given up resisting good impulses, and are willing to be "led by the spirit" (Rom. viii, 14). These are the sons of God in the highest sense.

It is better, however, to begin by thinking how the promise was fulfilled in our Lord's first disciples. "He shall guide you into all truth." It was a promise to the Church. Our Lord said the same thing rather more fully, earlier in the same discourse. "He shall teach you all things, and bring all things to your remembrance whatsoever I have said unto you" (John xiv, 26). The Apostles and their successors had to go forth and teach all nations, teaching them to observe all things whatsoever Christ had commanded. It was quite impossible that the first preachers of the gospel, poor uneducated men as they were, most of them, could go out into the world and lay the foundations of such a society as the Church has now become, without very special guidance. They had to remember all that Jesus had taught them (for the New Testament was not written). Next, they had to proclaim the glad tidings in such a manner as to make sure of its acceptance by vast multitudes, and with such a method as to secure the maintenance of the truth by congregations differing widely in kindred and language. So, for the Apostles, the guidance of the Holy Ghost was manifested, not merely in proclaiming, but in arranging, on a Catholic basis, the whole truth which Jesus had taught them orally. If they had been left to themselves they might have taught a good deal by the aid of unassisted memory, but they might have left out important parts of the Faith. The Holy Ghost was given to the Church to guide her above all things in selecting, and then in handing on to her children, the elements of truth most essential to salvation. So, although in the Gospels we have not nearly all the words which Jesus spoke while on earth, we may be quite sure that, in the New Testament, combined with the Creeds, we are possessed of the whole truth, or, as it is called by St. Jude (v. 3), "The faith which was once delivered unto the Saints."

---

"Good wine needs no bush:" and a hospital of all institutions least needs persuasive advocacy. All know what illness is. Some have been ill themselves, and know by experience either the need or the value of good advice and nursing. Some have seen others suffering from illness or accident: and these owe a debt of gratitude for their inexperience, which they may pay in part by a liberal gift to the hospital.—H. B.

